# Daily river flow simulation using ensemble disjoint aggregating M5-Prime model

**DOI:** 10.1016/j.heliyon.2024.e37965

**Published:** 2024-09-30

**Authors:** Khabat Khosravi, Nasrin Attar, Sayed M. Bateni, Changhyun Jun, Dongkyun Kim, Mir Jafar Sadegh Safari, Salim Heddam, Aitazaz Farooque, Soroush Abolfathi

**Affiliations:** a Canadian Centre for Climate Change and Adaptation, University of Prince Edward Island, Charlottetown, Canada; b Department of Statistical Sciences, University of Padova, Padova, Italy; c Department of Civil, Environmental and Construction Engineering and Water Resources Research Center, University of Hawaii at Manoa, Honolulu, HI, USA; d UNESCO-UNISA Africa Chair in Nanoscience and Nanotechnology College of Graduates Studies, University of South Africa, Muckleneuk Ridge, Pretoria, 392, South Africa; e School of Civil, Environmental and Architectural Engineering, College of Engineering, Korea University, Seoul, Republic of Korea; f Department of Civil and Environmental Engineering, Hongik University, Seoul, Republic of Korea; g Department of Geography and Environmental Studies, Toronto Metropolitan University, Toronto, Ontario, Canada; h Department of Civil Engineering, Yaşar University, Izmir, Turkey; i Faculty of Science, Agronomy Department, Hydraulics Division, Laboratory of Research in Biodiversity Interaction Ecosystem and Biotechnology, University 20 Août 1955, Route El Hadaik, BP 26, Skikda, Algeria; j Faculty of Sustainable Design Engineering, University of Prince Edward Island, Charlottetown, PE, Canada; k School of Engineering, University of Warwick, CV4 7AL, Coventry, UK

**Keywords:** River flow, Machine learning, Predictive model, Forecasting, M5P, Hybrid machine learning

## Abstract

Accurate prediction of daily river flow (*Q*_*t*_) remains a challenging yet essential task in hydrological modeling, particularly crucial for flood mitigation and water resource management. This study introduces an advanced M5 Prime (M5P) predictive model designed to estimate *Q*_*t*_ as well as one- and two-day-ahead river flow forecasts (i.e. *Q*_*t+1*_ and *Q*_*t+2*_). The predictive performance of M5P ensembles incorporating Bootstrap Aggregation (BA), Disjoint Aggregating (DA), Additive Regression (AR), Vote (V), Iterative classifier optimizer (ICO), Random Subspace (RS), and Rotation Forest (ROF) were comprehensively evaluated. The proposed models were applied to a case study data in Tuolumne County, US, using a dataset comprising measured precipitation (*P*_*t*_), evaporation (*E*_t_), and *Q*_*t*_. A wide range of input scenarios were explored for predicting *Q*_*t*_, *Q*_*t*+1,_ and *Q*_*t*+2_. Results indicate that *P*_*t*_ and *Q*_*t*_ significantly influence prediction accuracy. Notably, relying solely on the most correlated variable (e.g., *Q*_*t*-1_) does not guarantee robust prediction of *Q*_*t*_. However, extending the forecast horizon mitigates the influence of low-correlation input variables on model accuracy. Performance metrics indicate that the DA-M5P model achieves superior results, with Nash-Sutcliff Efficiency of 0.916 and root mean square error of 23 m^3^/s, followed by ROF-M5P, BA-M5P, AR-M5P, AR-M5P, RS-M5P, V-M5P, ICO-M5P, and the standalone M5P model. The ensemble M5P modeling framework enhanced the predictive capability of the stand-alone M5P algorithm by 1.2 %–22.6 %, underscoring its efficacy and potential for advancing hydrological forecasting.

## Graphical abstract

**Figure:**
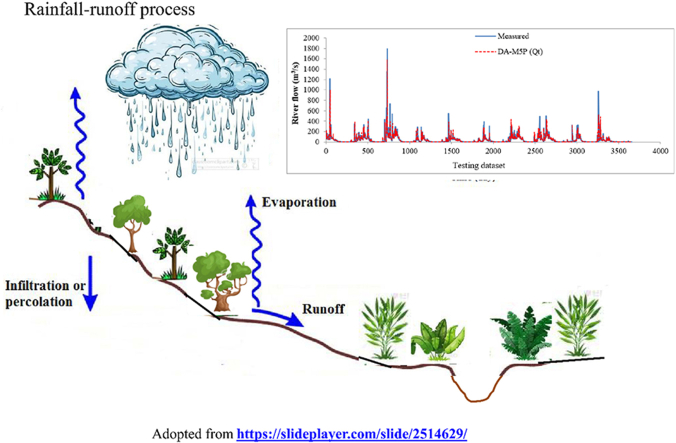

## 1 Introduction

Freshwater systems are one of the most precious natural resources on the planet, crucial to maintaining a balanced and sustainable socioeconomic growth, ecosystem health and function, as well as public health. A detailed understanding of the river flow (*Q*_*t*_) facilitates a wide range of applications spanning from drinking water supply and irrigation for agricultural lands, to generating hydropower renewable energies. However, the fluvial flow processes are highly complicated and influenced by several key factors, such as the topography of the watershed, vegetation cover, soil properties, channel characteristics, groundwaters, distribution of precipitation, and snowmelt [1,2].

Rainfall-runoff modeling has evolved over nearly five decades, beginning with early efforts to forecast discharge from rainfall using various regression methods [3,4]. There has also been a concerted effort to incorporate physical process knowledge and concepts into hydrological models [5]. A comprehensive review by Kirchner (2006) highlighted the effectiveness of these models in addressing spatial variations in watersheds' hydrological processes, physical characteristics, and boundary conditions. Recent advancements in computer technology, coupled with the availability of high-resolution spatial and temporal data, have significantly enhanced the development of sophisticated hydrological simulations [6].

In recent years, several early warning systems have been implemented to provide real-time measurements of river flows and associated hydrological parameters. This has facilitated the implementation of sustainable and effective surface water management strategies, helping to prevent disruptive flood inundations [7]. Fernández-Nóvoa et al. [8] developed MIDAS tools: A New Integrated Flood Early Warning System for the Miño River, by coupling a hydrologic (HEC-HMS) and a hydraulic (Iber+) models using precipitation forecast as input data. The design, operation, and maintenance of real-time remote sensing warning systems require significant and continual investment, which may not be feasible for communities in low-middle-income countries. In this context, it is necessary to develop robust, innovative, inexpensive, highly accurate, and reliable forecasting methods. Forecasting river flow is essential to address global water resource management challenges including reducing flooding, managing droughts, and planning new water management projects [[9], [10], [11], [12]].

Developing a robust predictive model for river flow is a difficult task due to the complex and non-linear behavior of river flows at both temporal and spatial scales, as well as the uncertainty associated with all the parameters influencing the flow. The complexities associated with the river flow predictive models increase as the temporal scale of forecasting increase from several days to months. The temporal scale of river flow prediction falls into two categories: long-term (i.e. s, weekly, monthly, yearly) and short-term (i.e., hourly or daily) forecasting. To this date, a number of studies adopted parametric and non-parametric modelling techniques to simulate river flows at different timeframes (e.g. weekly, monthly, seasonal, and yearly).

In parametric modeling approach, river flow data is assumed to follow a particular probability distribution, and the parameters of this distribution are estimated. The normal (Gaussian) distribution, log-normal distribution, gamma distribution, and Pearson type III distribution are some of the most commonly used parametric models for simulating river flows. When the data exhibit clear statistical patterns that can be well-explained by a known distribution, these parametric models prove to be useful. However, it is important to note that they require assumptions regarding the underlying distribution of the data.

In contrast, nonparametric modeling does not rely on any assumptions regarding the distribution of the data. These methods do not explicitly specify a probability distribution; instead, their design focuses on capturing patterns and relationships present in the data. Nonparametric models, being free from predetermined parameters, offer increased flexibility and adaptability to different types of data. They are particularly valuable in situations where the underlying distribution is unknown or when the data exhibit complex patterns that parametric models might not adequately capture.

Recently, process-based physical methodologies and data-driven modeling approaches have been explored for predicting river flows [13]. Process-based physical methods are advantageous, allowing for detailed understanding of the hydrological processes. However, process-based physical models can not readily generate ensembles and require extensive input data for the model setup, while data-driven models rely exclusively on input-output data and therefore have great potential for river flow forecasting. Data-driven approaches offers simplicity of model development and capability for real-time implementation, which make them efficient choice for predicting hydrological phenomena without the need for detailed understanding of the complicated underlying processes and extensive additional catchment information.

Applications of Artificial Intelligence (AI) methodologies in addressing complex multi-faceted environmental hydraulics and hydrological problems have been dramatically increased in recent years [[14], [15], [16], [17], [18], [19], [20]]. The AI and time-series models are adopted for forecasting river flows [[21], [22], [23]]. Machine learning models (ML) are a subset of AI techniques, capable of robust forecasting by self-learning from physical processes. ML techniques are mainly categorized into supervised and unsupervised methods. Supervised MLs include classification, regression, and optimization methods, whereas unsupervised MLs include clustering methods. Artificial Neural Network (ANN), Support Vector Machine/Regression (SVM/SVR), adaptive neuro-fuzzy inference system (ANFIS), Extreme Learning Machine (ELM), Genetic Programming (GP), and group method data handling (GMDH) models are the most commonly used methods in water sciences, especially for forecasting river flow [[24], [25], [26], [27], [28], [29], [30]].

Modaresi et al. [31] utilized K-Nearest Neighbor Regression (KNN), ANN, Least Square-Support Vector Regression (LS-SVR), and Generalized Regression Neural Network (GRNN) methods to forecast monthly river flow data. They found that models performed differently considering linear and non-linear conditions. For the linear conditions, ANNs yield the best performance, while for the non-linear conditions, LS-SVR, GRNN, and KNN showed a more robust performance [31]. Long Short-Term Memory (LSTM) deep learning model is adopted for river flow modeling for a case study of Hackensack River Watershed in New Jersey, USA [32], the Western United States' rivers [33], and the Yarkant River, Northwest China [34]. Singh et al. [35] applied Gaussian linear regression model (GLM), Gaussian generalized additive model (GAM), multivariate adaptive regression splines (MARSs), ANN, random forest (RF), and 1D convolutional neural network (1D-CNN) for river flow prediction over the Sutlej River basin in the western Himalaya for the 2041–2070 and 2071–2100 periods. Their findings suggest that river flow will increase during the monsoon season in the 2050s and 2080s under SSP585 and SSP245 emission scenarios but decrease during the pre-monsoon, post-monsoon, and winter.

Analysis of ML-based forecasting models show that stand-alone models are incapable of robust simulation of hydrological time series (e.g., river flow), specifically for the case of capturing extreme values [13,36]. Despite significant research efforts to improve the precision of the stand-alone ML models, achieving accurate forecasts remains challenging. Hybrid models can offer computational power and reliability to enhance forecasting for hydrological problems. In hydrology, different approaches including metaheuristic optimization [37], decomposition [38,39], and ensemble methods (e.g., bagging) [40] are developed to advance the hybrid-ML modeling.

Adamowski and sun [41] combined wavelet transforms (WA) and ANN as a hybrid method (i.e. WA-ANN) for forecasting river flows at two rivers in Cyprus (Kargotis at Evrychou and Xeros at Lazarides) for lead-times of one and three days. For both cases, the coupled WA-ANN model produced more accurate short-term river flow forecasts than ANNs. Optimally Pruned Extreme Learning Machine (OPELM) and Chi-Square Automatic Interaction Detector (CHAID) techniques were used by Attar et al. [1] for modeling the deterministic part of the river flow equation at monthly time-step, while Autoregressive Conditional Heteroskedasticity (ARCH) was adopted for the stochastic part of the flow equation. Khazaee Poul et al. [42] utilized multi-linear regression (MLR) as a statistical method and ANN, KNN, and ANFIS as non-parametric regression methods for monthly river flow modeling in the St. Clair River. The results show that while adding lag times for river flow, temperature, and precipitation significantly improve the accuracy of predictions, the hybrid modelling approach outperforms the stand-alone ML-based models. Adnan et al. [43] implemented extreme learning machine (ELM)-based models integrated with metaheuristic methods, including Mayfly optimization algorithm (MOA), Particle Swarm Optimization (PSO), simulated annealing (SA), and Grey wolf optimization (GWO) for monthly river flow prediction. Based on their finding, ELM–PSO-GWO outperforms ELM, ELM–PSO, ELM–MOA, and ELM–SAMOA by reducing RMSE by 20 %, 5.1 %, 6.2 %, and 4.6 %, respectively, during the testing stage.

Khosravi et al. [36] implemented reduced error pruning tree (REPT) model hybridized with bootstrap aggregation-Bagging (BA), random committee (RC), random subspace (RS), additive regression (AR) and disjoint aggregating-Dagging (DA) to investigate optimum method for river flow modeling in Iran. The findings confirmed that all hybrid models tested increased the prediction accuracy of the REPT model. Khosravi et al. [44] applied optimized deep learning convolutional neural network (CNN) using BAT metaheuristic algorithm (i.e. CNN-BAT) for daily river flow prediction in the Korkorsar catchment, Iran, and show that the proposed CNN-BAT outperform Multi-layer perceptron (MLP)-BAT, ANFIS-BAT, SVR-BAT, and RF-BAT. Optimization problems can help resolve the challenge of obtaining accurate weights for hyperparameter tuning. Decomposition techniques can enhance modeling performance as an effective data-preprocessing method. Meanwhile, ensemble models have the capability to improve overall model performance by leveraging the benefits of two coupled models. Granata and Nunno [45] implemented MLP, radial basis function (RBF)-ANN, LSTM, and Bidirectional-LSTM for daily river flow forecasting in the UK. Their study found that all developed models provided accurate predictions in the short term (1–3 days ahead), with the RBF-ANN often outperforming other algorithms in medium-term forecasts (7–15 days ahead). Santos et al. [46] applied wavelet neural networks (WNN) for river flow forecasting at both short-term (daily) and long-term (weekly, fortnightly, and monthly) intervals in the Mahanadi River basin, India. They reported that the WNN approach demonstrated good performance (Nash-Sutcliffe Efficiency (NSE) ranging from 0.299 to 0.987 across all time horizons and stations), particularly for long-term forecasts, indicating its potential viability for other catchments.

Granata et al. [47] developed a novel additive regression (AR) model, including AR-RBF and the Pace Regression of the Multilayer Perceptron and Random Forest (MLP-RF-PR), for river flow forecasting in German rivers. Their findings suggest that both AR-RBF and MLP-RF-PR are reliable forecasting tools. Jemei et al. [48] developed and compared the predictive power of several models, including the Convolutional Neural Network-Bidirectional Gate Recurrent Unit (CNN-BiGRU), CNN-Bidirectional Recurrent Neural Network, Random Variational Function Link (RVFL), Kernel Extreme Machine Learning (KELM), and CNN-BiGRU for streamflow prediction in Prince Edward Island (PEI), Canada. They concluded that the MVM-CNN-BiGRU model outperformed the other hybrid models.

## 2 research significance

Despite many research investigations dedicated to ML-based modelling, there is no conclusive evidence that the ML technique would be the most appropriate and robust method for hydrological problems. This study investigates the robustness and appropriateness of stand-alone M5 Prime (M5P) and its ensemble versions for river flow prediction (*Q*_*t*_), and one- and two-day-ahead forecasting (Q_t+1_ and Q_t+2_). For the first time, a novel M5P ensemble incorporating Bootstrap Aggregation (BA), Disjoint Aggregating (DA), Additive Regression (AR), Vote (V), Iterative classifier optimizer (ICO), Random Subspace (RS), and Rotation Forest (ROF) algorithms is developed for river flow prediction (*Q*_*t*_, *Q*_*t+1*_, and *Q*_*t+2*_). Detailed statistical analyses are conducted to examine the accuracy and efficiency of the stand-alone and ensemble M5P models in river flow simulation. Further analysis is performed to identify which combination of input variables has the highest influence on the river flow predictions.

## 3 Study area

**Fig. 1:**
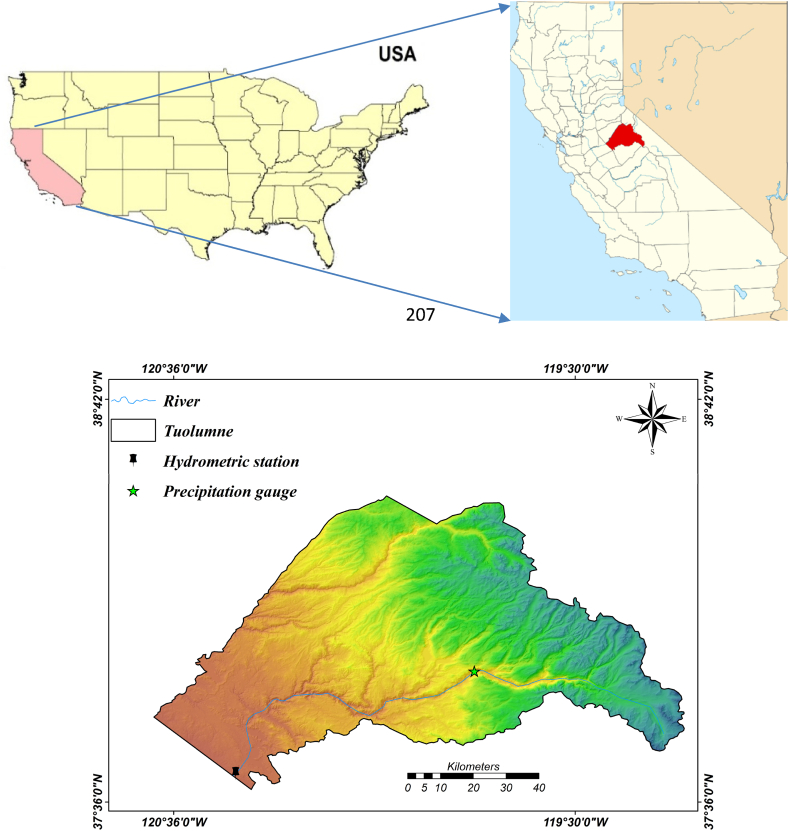
The study area, Tuolumne catchment, and the location of hydrological sensing stations.

This study leverages an extensive hydrological dataset, which provides insights into local climate variability as well as inter-annual and seasonal climate fluctuations. Tuolumne County, California, was chosen as the study catchment area, encompassing the watershed of USGS 11284400 BIG C AB WHITES GULCH NR GROVELAND, CA. The hydrologic unit code for this region is 18040009, with coordinates of Latitude 37°50′31″ and Longitude 120°11′02". Tuolumne County covers a total area of 2274 square miles (5890 km^2^), with 2221 square miles (5750 km^2^) designated as land and 54 square miles (140 km^2^), or 2.4 %, as water. The specific study area features a drainage region of 42.45 km^2^ (16.4 mi^2^), as illustrated in Fig. 1 (US Gazetteer files: 2010, 2000, and 1990).

Situated in California, USA, this region enjoys abundant sunshine, averaging 325 sunny days per year. The climate is characterized as moderate, with an annual mean temperature of 60 °F. Rainfall is relatively high, with an annual average of 32.85 inches. Elevation within the study area ranges from 1150 to 3999 m above sea level (m.a.s.l.). The watershed is primarily managed by the O'Shaughnessy Dam, which serves as the key outlet. This dam is vital for supplying drinking water and generating hydropower for nearly 3 million residents [49].

## 4 Methodology

**Fig. 2:**
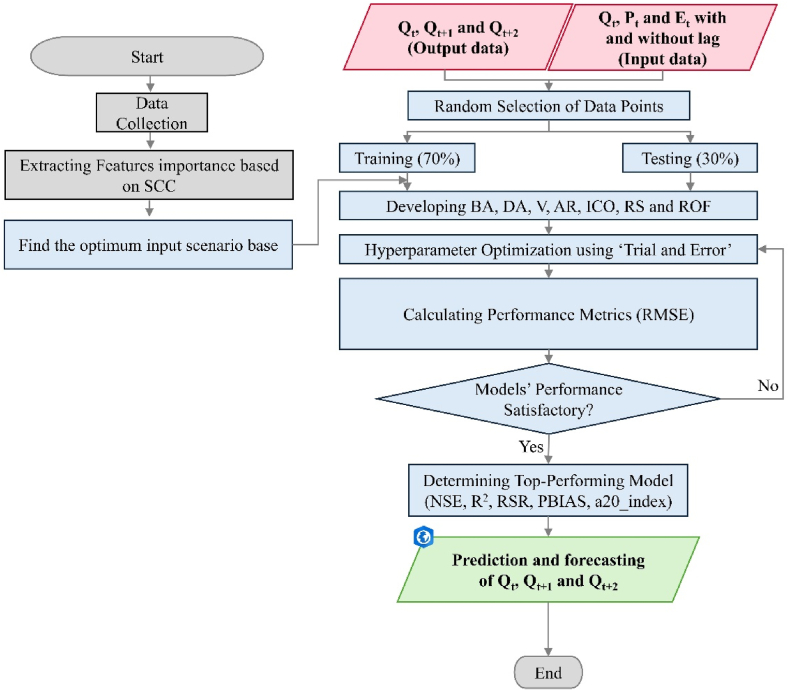
Flowchart of the proposed methodological framework for river flow forecasting.

The methodological framework proposed for robust river flow modeling based on readily available environmental sensing data and advanced tree-based machine learning. Techniques is illustrated in Fig. 2.

### 4.1 Data collection and sample size

**Fig. 3:**
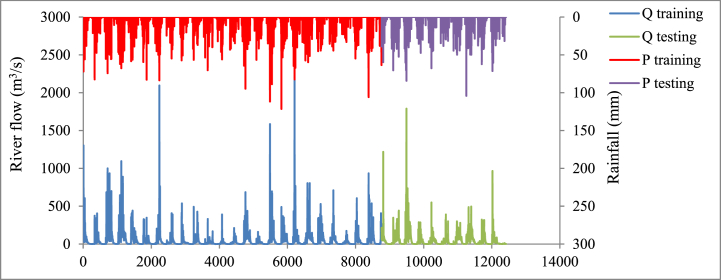
Precipitation and river flow variables during training and testing dataset.

This study adopts ∼33 years of measured data at Tuolumne County, California, USA. The dataset includes precipitation (*P*_*t*_), evaporation (E), and natural *Q*_*t*_ (i.e. based on National Hydrography Plus (NHD+) database), from 1980 to 2013 (Fig. 3). *Q*_*t*_ database can be accessed at the USGS website (https://waterdata.usgs.gov/monitoring-location/11284400/). The Daymet database was utilized for the rest of the parameters used in this study. Data from July 1, 1980 to December 31, 2003 (8761 data points) was employed for model development (i.e. training dataset), and the rest of the data from January 1, 2004 to December 31, 2013 (3653 data points) was utilized for the model evaluation (i.e. test dataset). Although there is no universal guideline on the appropriate ratio of train-test data split, the 70:30 ratio is the most commonly validated for ML modeling studies. An overview of the input variables used for river flow prediction and statistical analysis of the data can be found in Table 1, Table 2.

**Table 1:** Description of the input data utilized for rive-flow modelling.

***Attribute***	***Description***	***Unit***
*P*_*t*_	Average daily precipitation	mm/day
*E*_*t*_	Average evaporation	mm/day
*Q*_*t*_	Average daily river flow	cfs

**Table 2:** Statistical analysis of the dataset parameters used in model training and evaluation.

Training	*P*_*t*_	*E*_*t*_	*Q*_*t*_
Min	0.00	89.99	0.00
Max	86.83	2050.70	1370.00
Mean	2.89	486.30	10.38
Std	9.06	226.70	51.13
Testing	*P*_*t*_	*E*_*t*_	*Q*_*t*_
Min	0.00	108.70	0.00
Max	82.63	2100.5	752.00
Mean	2.46	521.33	7.57
Std	7.76	244.00	37.73

### 4.2 Input/output formulation

**Fig. 4:**
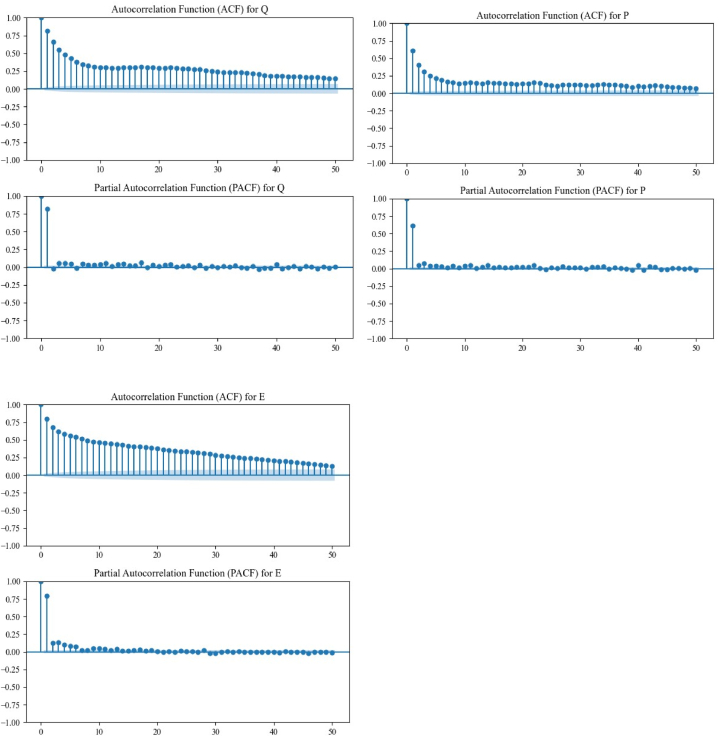
ACF and PACF for determining the significant lags for *Q*, *P*, and *E* variables at 5 % confidence interval.

Input variables including, *P*_*t*_, *P*_*t*_ with lag-time (i.e. *P*_*t-1*_*, P*_*t-2*_), E_t_ and *Q*_*t*_ with lag-time are the highly effective parameters in river flow modeling. To determine the effective lag time of each variable and construct different input combination scenarios, we employed the autocorrelation function (ACF), partial autocorrelation function (PACF), and Spearman Correlation Coefficient (SCC) in the model evaluation (Fig. 4). First, the appropriate lag number is determined, and then, the potential input variable with the highest SCC was selected to evaluate the correlation between the SSC and the accuracy of *Q*_*t*_ simulations. Ten different input combination scenarios were constructed from the input variables (Table 1). Scenario 1 to 4 only considers the precipitation data as model input, whereas scenario 5 and 6 combine precipitation and river flow parameters. Scenarios 7 and 8 combine precipitation and temperature parameters as the input feed, scenario nine only considers the *Q*_*t*_ data with diffident lag-time, and scenario 10 combines precipitation, temperature, and river flow parameters as the input (Table 3). To find the most effective and reliable input combination scenario, all of the scenarios described in Table 1 were fed to the developed M5P-based models, and then the modeling results were compared in terms of Root Mean Square Error (RMSE) to determine the most efficient input combination scenario. The input scenario leading to the lowest RMSE is selected as the best modeling configuration.

**Table 3:** Summary of input combination scenarios assessed for the prediction of *Q*_*t*_, *Q*_*t+1*,_ and *Q*_*t+2*_.

No.	potential input scenario	Output	Potential input scenario	Output
1	*P*_*t*_	*Q*_*t*_	*P*_*t*_	*Q*_*t+ 1*_ and *Q*_*t+2*_
2	*P*_*t*_*, P*_*t-1*_	*Q*_*t*_	*P*_*t*_*, P*_*t-1*_	*Q*_*t+ 1*_ and *Q*_*t+2*_
3	*P*_*t*_*, P*_*t-1*_*, P*_*t-2*_	*Q*_*t*_	*P*_*t*_*, P*_*t-1*_*, P*_*t-2*_	*Q*_*t+ 1*_ and *Q*_*t+2*_
4	*P*_*t*_*, P*_*t-1*_*, P*_*t-2*_*, P*_*t-3*_	*Q*_*t*_	*P*_*t*_*, P*_*t-1*_*, P*_*t-2*_*, P*_*t-3*_	*Q*_*t+ 1*_ and *Q*_*t+2*_
5	*P*_*t*_*, Q*_*t-1*_	*Q*_*t*_	*P*_*t*_*, Q*_*t*_	*Q*_*t+ 1*_ and *Q*_*t+2*_
6	*P*_*t*_*, P*_*t-1*_*, Q*_*t-1*_	*Q*_*t*_	*P*_*t*_*, P*_*t-1*_*, Q*_*t*_*, Q*_*t-1*_	*Q*_*t+ 1*_ and *Q*_*t+2*_
7	*P*_*t*_*,* E_t_	*Q*_*t*_	*P*_*t*_*,* E_t_	*Q*_*t+ 1*_ and *Q*_*t+2*_
8	*P*_*t*_*, P*_*t-1*_*, P*_*t-2*_*, P*_*t-3*_*,* E_t_, E_t-1_, E_t-2_, E_t-3_	*Q*_*t*_	P_t_, P_t-1_, P_t-2_, P_t-3_, E_t_, E_t-1_, E_t-2_, E_t-3_	*Q*_*t+ 1*_ and *Q*_*t+2*_
9	*Q*_*t-1*_*, Q*_*t-2*_*, Q*_*t-3*_	*Q*_*t*_	*Q*_*t*_*, Q*_*t-1*_*, Q*_*t-2*_*, Q*_*t-3*_	*Q*_*t+ 1*_ and *Q*_*t+2*_
10	P_t_, P_t-1_, P_t-2_, P_t-3_, E_t_, E_t-1_, E_t-2_, E_t-3_, Q_t-1_, Q_t-2_, Q_t-3_	*Q*_*t*_	P_t_, P_t-1_, P_t-2_, P_t-3_, E_t_, E_t-1_, E_t-2_, E_t-3_, Q_t_, Q_t-1_, Q_t-2_, Q_t-3_	*Q*_*t+ 1*_ and *Q*_*t+2*_

### 4.3 Model development

Several criteria, including data quality, data splitting ratio, model prediction power, and model design, can significantly influence the performance of ML-based modeling. Determining the optimum values of the model's hyperparameters is crucial for appropriately designing the model structure. The optimum values of the model's hyperparameter are case-specific and vary across different studies. As such, identifying the optimum value will enhance the model's performance.

In this study, initially, the default values were considered for developing M5P models. Then, a trial-and-error approach was implemented to find the optimum value for all the model's variables. RMSE was used to find the efficient optimum values of the model's hyperparameter (i.e., lower RMSE). Waikato Environment for Knowledge Analysis (WEKA 3.9) was utilized for model development and optimization.

### 4.4 Model description

#### 4.4.1 M5 Prime (M5P)

The M5 Prime (M5P) is an improved version of the original M5Tree proposed by Quinlan [50], for which a combination of the decision tree (DT) and a simple regressions model (LR) at the leaf nodes was proposed [[51], [52], [53], [54]]. Thus, the regression tree (RT) with leaves suits multiple linear regression (MLR) models rather than discrete values. In addition, the main criterion for the selection of nodes is reduction in the error as a function of the standard deviation of the calculated data [[55], [56], [57]]. Building an M5P model needs three steps including (*i*) an ensemble of RT models is generated from the training dataset, and an LR model is created for each node of the constructed RT, (*ii*) the so-called "*first post-pruning*" stage is made by removing the nodes whose contribution to the error reduction is marginal, and (*iii*) the so-called "*second post-pruning*" stage is adopted for reducing the number of RT and simultaneously maintaining the competitiveness and the accuracy of the model [58].

#### 4.4.2 Bootstrap aggregation

Bootstrap aggregation (BA) is amongst the ensemble-learning algorithms. Breiman [59] proposed the BA algorithm by combining the bootstrap and aggregation concepts. The Bootstrap method is sampling with replacement, used for randomly creating an ensemble of sub-samples for the training dataset to overcome the instability. Consequently, each created sub-sample becomes more specialized for one base learner and eventually trains it. The final response of the model is calculated as majority voting for classification and averaging for regression. Furthermore, the output of the final BA model can be determined as follow:

(1) $$y=\frac{\sum_{i=1}^{N}y_{i}}{N}$$

where *y*_*i*_ is the single response provided by a single base-learner corresponding to the ith model, *y* is the calculated average response of the final model, and *N* is the number of created base learners [60,61].

#### 4.4.3 Disjoint aggregating

Disjoint Aggregating (DA), also known as Dagging, is one of the most adopted ensembles learning algorithms for regression or classification problems [62]. Regarding the overall mathematical formulation, Dagging is similar to the Bagging algorithm, but instead of Bootstrap, DA uses disjoint sampling. DA divides the training dataset into *k* parallel subsets, creating a base-learn model for each subset. The final DA response is calculated using majority voting. Additionally, DA only allows for one noisy pattern in each subset, while there is no need for extra computational resources because the same number of examples is used as the training subset [63,64].

#### 4.4.4 Additive regression

Hastie and Tibshirani [65] first introduced the additive regression model (AR). If a linear model is to be developed from an ensemble of independent variables, i.e., *x*, *z*, and *s*, the effect of each independent variable is examined separately in the absence of interactions using the AR algorithm as follows:

(2) $$Y_{t}=\beta_{0}+f\left(x_{t}\right)+g\left(z_{t}\right)+p\left(s_{t}\right)+u_{t}$$

where f,g,andp are arbitrary functions for each independent variable, $\beta_{0}$ is the intercept, and $u_{t}$ is the error. Hastie and Tibshirani [65] have proposed the so-called back fitting algorithm (BF) as an iterative algorithm. Suppose we need to estimate "*g"*, the BF algorithm estimates the "*f*" form "*g*" and vice versa until a suitable convergence is obtained [66].

#### 4.4.5 The vote algorithm

The voting algorithm (VA) is utilized to arbitrate among the response of several variant results, and the final response is selected as the voter output. The VA does not rely on the availability of a large dataset for training or any prior knowledge of the single base model. The individual variants can produce no or wrong results, leading to a very poor final response. A combination of simultaneous, related, or unrelated errors can cause incorrect response. The VA can be considered for selecting the best subset of a weak model using feature selection algorithms, a weighting strategy to the votes, or by adopting nearest neighbors [67,68].

#### 4.4.6 Iterative classifier optimizer

The iterative classifier optimizer (ICO) algorithm uses cross-validation to optimize the number of iterations for the classifier. The ICO optimizes a predefined classifier's total number of iterations by choosing a percentage split. The algorithm processes summarize as (*i*) mean calculation is adopted rather than a single estimation from pooled calculations; (*ii*) the total number of runs are selected for cross-validation; (*iii*) the optimization is based on an index metric, i.e., the root mean squared error (RMSE); and (*iv*) the default classifiers LogitBoost or AdaBoost are selected as base classifier. Overall, the ICO is performed in two main steps, starting by comparing the observed and predicted data in response to several models running and concluding by sending back the obtained response to the model for more learning and optimizing the final obtained results [69].

#### 4.4.7 Random subspace

Random sub-space (RS) proposed by Ho et al. [70] is a random sampling algorithm with the overall objective of improving weak classifiers and enhancing their accuracy. Using the RS algorithm, a low-dimensional sub-space can be obtained by randomly sampling initial high-dimensional features, and it takes advantage of the simultaneous combination of bootstrapping and aggregation. The RS algorithm can be summarized as (*i*) a low dimensional random subspace is obtained from the original feature space, using a random sampling strategy; (*ii*) for each low random sub-space obtained, a classifier is constructed; and (*iii*) the obtained results from each classifier are combined. RS algorithm is robust in improving the accuracy of classification and regression by taking advantage of the best features in the presence of a large number of features [71,72].

#### 4.4.8 Rotation forest

The rotation forest (ROF) algorithm is developed by Rodriguez et al. [73]. ROF is a multiple-classifier system using a bootstrap sampling strategy for generating a suite of weak classifiers based on a multivariate statistical technique, i.e., the principal component analysis (PCA). The ROF provides the best single classifier [74]. The PCA removes the correlation between features and then provides a suite of linear transformations (LtF) combined for building a randomized training dataset. Similar to the original random forest (RF), a single decision tree takes one individual randomized training set on board, and the final response is calculated using a majority vote. The success of the ROF is mainly due to the use of the '*rotation matrix*' for LtF [75,76].

**Fig. 5:**
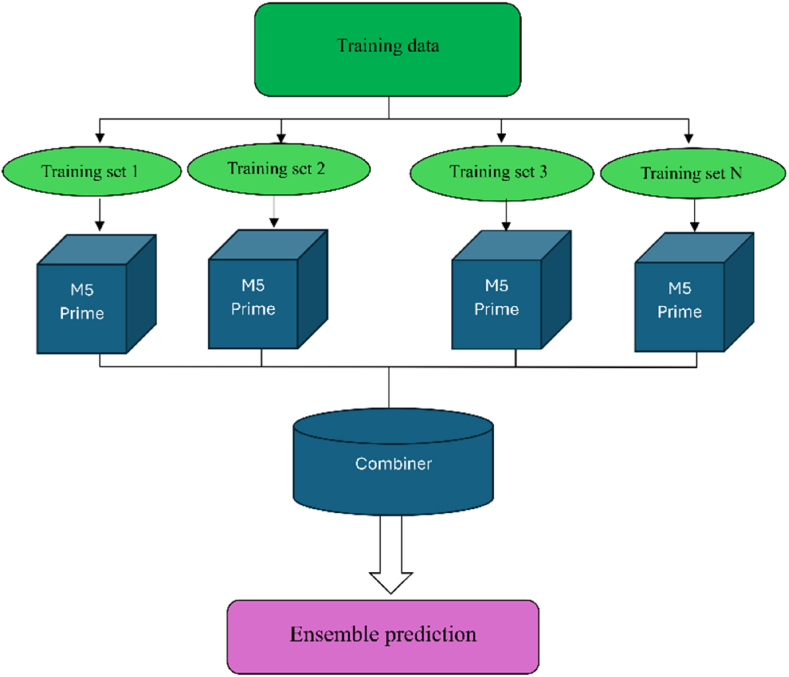
Methodological flowchart of the ensemble technique approach proposed in this study.

It's useful to illustrate how each ensemble-based model integrates with M5 Prime-based models, as shown in Fig. 5.

### 4.5 Model performance evaluation

This study provides an in-depth evaluation of the developed ML-based models' performance using visual (scatter plot, time-variation graph, and violin plot) and quantitative (RMSE, coefficient of determination, Nash-Sutcliff efficiency, Percent of BIAS, and RMSE-observations standard deviation ratio) performance indicators, and a20-index reliability assessment. The quantitative metrics adopted in this study are as follows [[77], [78], [79], [80]]:

(3) $$R^{2}=1-\left(\frac{\sum_{i=1}^{N}\left(Q_{t}^{Obs}-{\bar{Q}}_{t}^{Obs}\right)\left(Q_{t}^{\mathrm{Pr}\;e}-{\bar{Q}}_{t}^{\mathrm{Pr}\;e}\right)}{\sqrt{\sum_{i=1}^{N}{\left(Q_{t}^{Obs}-{\bar{Q}}_{t}^{Obs}\right)}^{2}}\sqrt{\sum_{i=1}^{N}{\left(Q_{t}^{\mathrm{Pr}\;e}-{\bar{Q}}_{t}^{\mathrm{Pr}\;e}\right)}^{2}}}\right)$$

(4) $$RMSE=\sqrt{\frac{\sum_{i=1}^{N}{\left(Q_{t}^{\mathrm{Pr}\;e}-Q_{t}^{Obs}\right)}^{2}}{N}}$$

(5) $$NSE=1-\sqrt{\frac{\sum_{i=1}^{N}{\left(Q_{t}^{\mathrm{Pr}\;e}-Q_{t}^{Obs}\right)}^{2}}{\sum_{i=1}^{N}{\left(Q_{t}^{Obs}-{\bar{Q}}_{t}^{Obs}\right)}^{2}}}$$

(6) $$PBIAS=\sqrt{\frac{\sum_{i=1}^{N}\left(Q_{t}^{\mathrm{Pr}\;e}-Q_{t}^{Obs}\right)}{\sum_{i=1}^{N}Q_{t}^{Obs}}}$$

(7) $$RSR=\frac{RMSE}{STDEV_{obs}}=\sqrt{\frac{\sum_{i=1}^{N}{\left(Q_{t}^{\mathrm{Pr}\;e}-Q_{t}^{Obs}\right)}^{2}}{\sum_{i=1}^{N}{\left(Q_{t}^{Obs}-{\bar{Q}}_{t}^{Obs}\right)}^{2}}}$$

(8) $$a20\_index=\frac{m20}{N}$$

where $Q_{t}^{\mathrm{Pr}\;e}$ is the predicted river flow value, $Q_{t}^{Obs}$ is the observed river flow value, and are the mean observed and predicted river flow value, respectively, and *N* is the dataset number. The metric *m*_20_ represents the number of samples for which the ratio of observed/predicted values fall between 0.80 and 1.20. This study utilized RMSE as a robust error metric due to unit compatibility with the target variable, allowing for a readily interpretation of the results and understanding of the difference. NSE offers a dimensionless measure, facilitating comparison between different models and datasets. PBIAS metric is utilized to identify the model overestimation and underestimation.

Furthermore, the combination of PBIAS and RSR allows for a qualitative classification of model evaluation, providing valuable information on the model's overall performance. The implementation of wide range of quantitative statistical measures, following the guidelines by Barbosa et al. [81] (2019), enhances the comprehensiveness of the evaluation process (see Table 4).

**Table 4:** Model performance evaluation rating considered in this study.

Performance rating	RMSE	a-20_index	R^2^	NSE	PBIAS	RSR
Very good	The lower the RMSE, the model with higher performance		0.75< R^2^ ≤ 1.00	0.75<NSE≤1.00	PBIAS<±10	0.00≤RSR≤0.50
Good		For the ideal model is equal to 1.	0.65< R^2^ ≤ 0.75	0.65<NSE≤0.75	±10≤PBIAS<±15	0.50<RSR≤0.60
Satisfactory			0.50< R^2^ ≤ 0.65	0.50<NSE≤0.65	±15≤PBIAS<±25	0.60<RSR≤0.70
Unsatisfactory			R^2^ ≤ 0.50	NSE≤0.50	PBIAS≥±25	RSR>0.70

## 5 Results and analysis

### 5.1 Relative importance of input variables

**Fig. 6:**
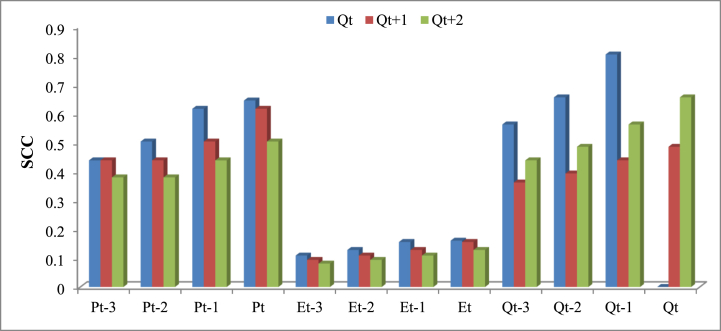
Relative importance of potential input variables.

The effectiveness of input variables on the river flow simulation is examined based on the SSC (Fig. 6). Based on the SSC, *Q*_*t-1*_ has the highest impact on *Q*_*t*_ prediction, followed by *Q*_*t-2*_, *P*_*t*_, *P*_*t-1*_, *Q*_*t-3*_*, P*_*t-2*_*, P*_*t-3*_*, E*_*t*_*, E*_*t-1*_*, E*_*t-2*_, and *E*_*t-3*_, respectively. For the *Q*_*t+1*_ forecasting, *P*_*t*_ is the most effective variable, followed by *P*_*t-1*_*, Q*_*t*_*, P*_*t-2*_*, P*_*t-3*_*, Q*_*t-1*_*, Q*_*t-2*_*, Q*_*t-3*_*, E*_*t*_*, E*_*t-1*_*, E*_*t-2*_*, E*_*t-3*_, respectively, while *Q*_*t*_ has the highest impact on *Q*_*t+2*_ forecasting. Overall, the analysis of SCC results shows that river flow with lag time is more important than precipitation with and without lag time. The evaporation data has the least influence on the modeling performance. In addition, findings revealed that increasing the number of days lag-time (e.g., from 1 to 3 days) reduces the effectiveness of forecasting.

### 5.2 Best input scenario

Analysis of SCC (Table 5) and RMSE scores of the M5P-based models (Tables 6 and 7) shows that models the input combination scenarios No.10, No. 6, and No. 5 provide the most effective input combination scenarios for *Q*_*t*_, *Q*_*t+1*,_ and *Q*_*t+2*_ forecasting, respectively. The results show that the input scenario with all 11 input variables (Scenario No. 10) achieved the highest *Q*_*t*_ prediction accuracy.

**Table 5:**
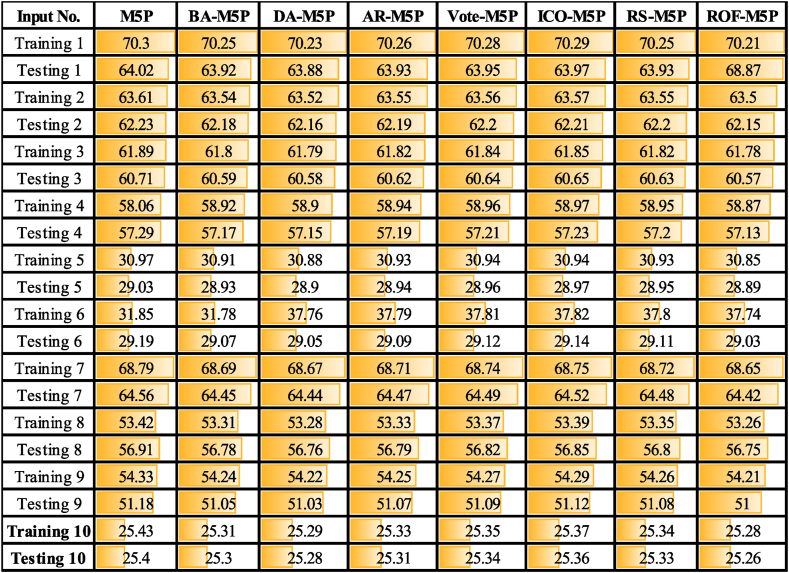
Comparison of ML models predictive performance for *Q*_*t*_ across different input scenarios based on RMSE metric.

**Table 6:**
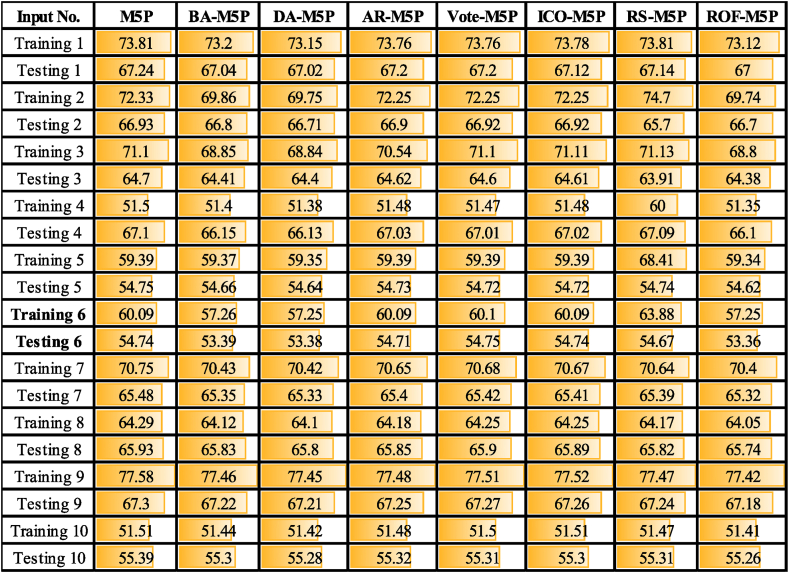
Comparison of ML models predictive performance for *Q*_*t+1*_ across different input scenarios based on RMSE metric.

**Table 7:**
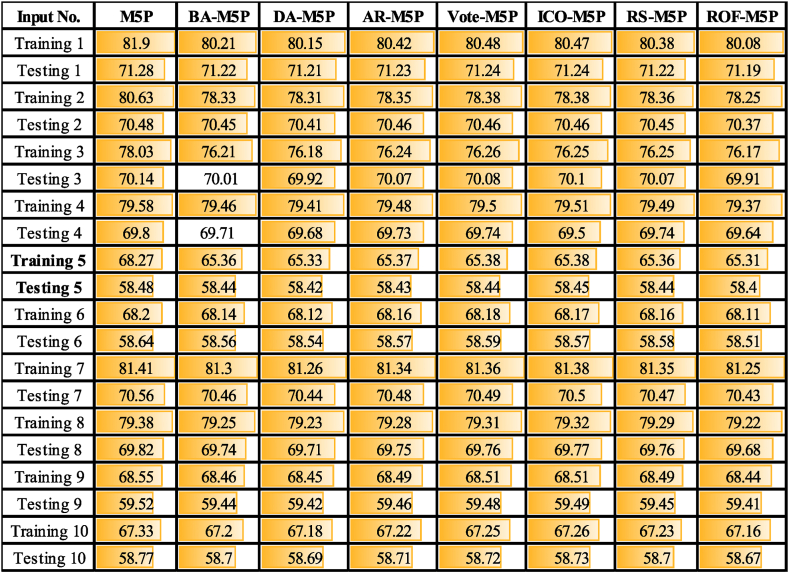
Comparison of ML models predictive performance for *Q*_*t+2*_ across different input scenarios based on RMSE metric.

Analysis of the results (Table 3, Table 4, Table 5) shows that by including the P_t-1_ to the inputs, 2.8 %, 0.46 %, and 1.12 %, the predictive model performance is increased for *Q*_*t*_*, Q*_*t+1*,_ and *Q*_*t+2,*_ respectively. Inclusion of *P*_*t-2*_ resulted in 2.44 % (*Q*_*t*_), 3.77 % (*Q*_*t+1*_), and 1.6 % (*Q*_*t+2*_) enhanced accuracy of predictions, while incorporating *P*_*t-3*_ resulted in 5.63 % (*Q*_*t*_), 0.2 % (*Q*_*t+1*_), and 2.07 % (*Q*_*t+2*_). The addition of *E*_*t*_ to the modeling process (compare input scenario No.7 and No.1) resulted in reducing the M5P models' performance by approximately 6.93 % for *Q*_*t*,_ while this parameter helped to improve the modeling predictions for *Q*_*t+1*_ (2.6 %) and *Q*_*t+2*_ (1 %). Removing the *Q*_*t*_*, Q*_*t-1*_, *Q*_*t-2*,_ and *Q*_*t-3*_ from input parameters (comparing scenarios No.10 and No.8) has led to a reduction of the modeling accuracy by 55.36 %, 5.6 %, and 15.85 % for *Q*_*t*_, *Q*_*t+1*,_ and *Q*_*t+2*,_ respectively. Removing the *P*_*t*_ and *E*_*t*_ variables with and without lag-time (compare scenarios No.10 and No.9) reduced the prediction performance by 50.37 %, 3.60 %, and 14.75 % for *Q*_*t*_, *Q*_*t+1*,_ and *Q*_*t+2*_, respectively.

### 5.3 Model evaluation and comparison

**Fig. 7:**
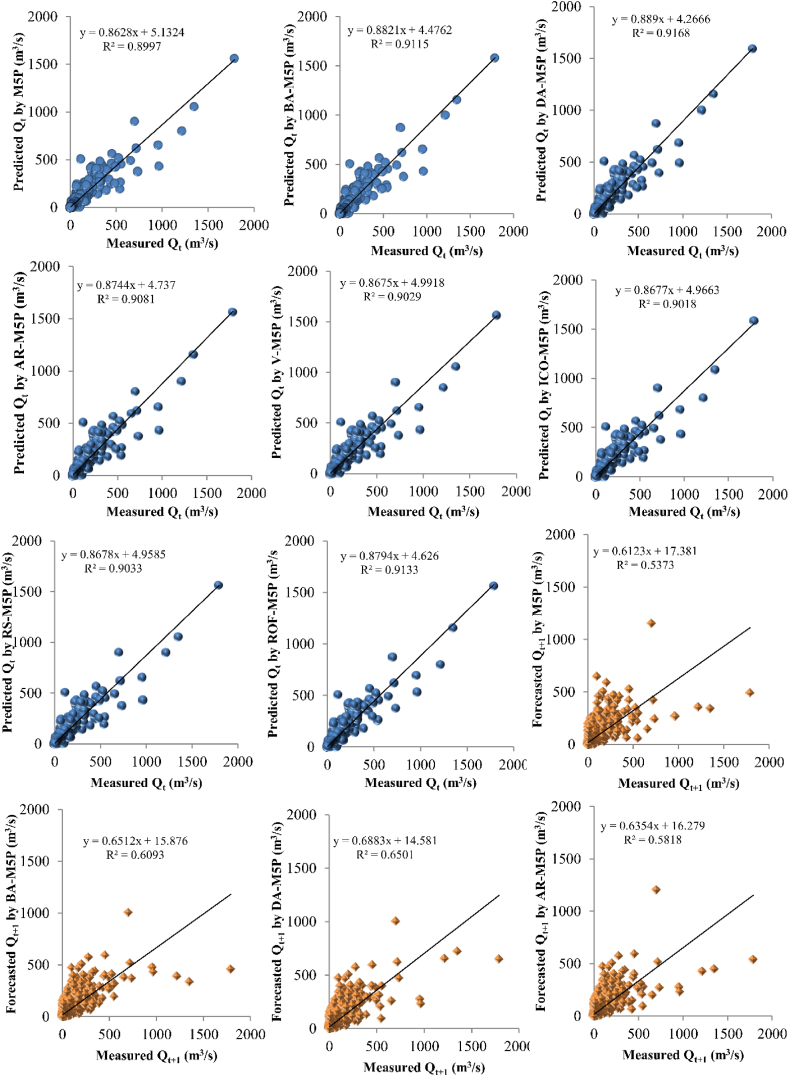

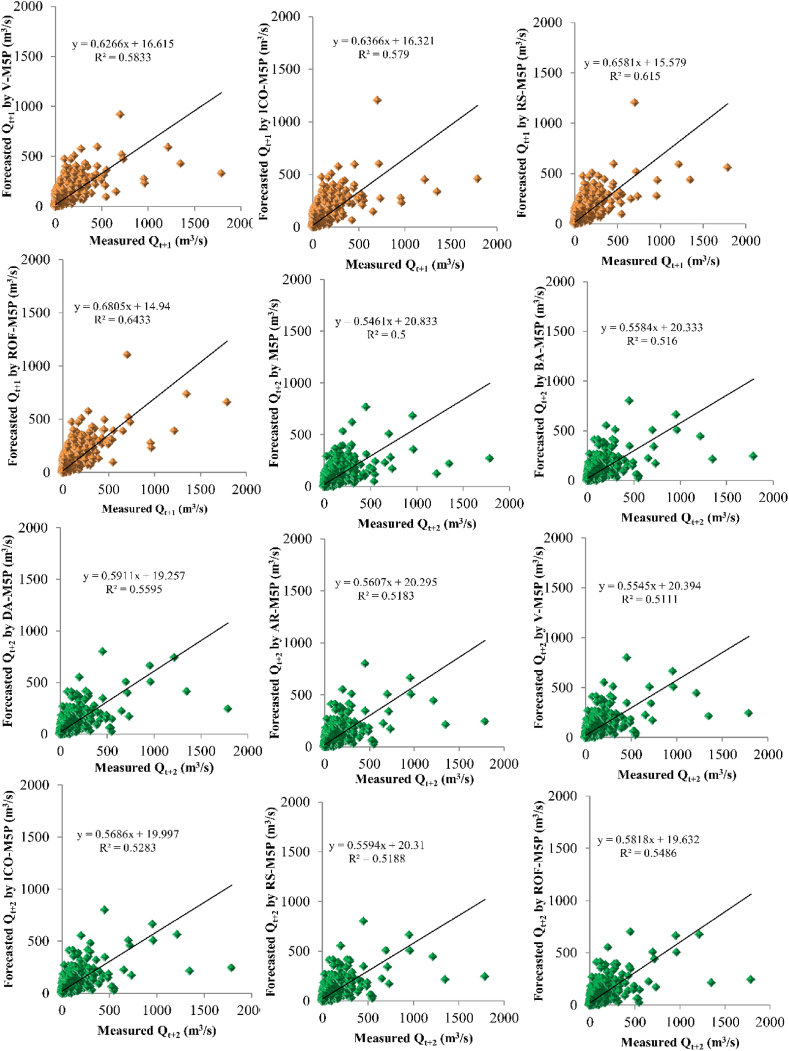
Scatter plot of predictive models performance for *Q*, *Q*_*t+1*_, and *Q*_*t+2*_ forecasting.

The prediction results are compared with the measured data, and the coefficient of determination (*R*^*2*^) was determined for all the developed models (Fig. 7). The results confirm robust performance for all the developed models, with R^2^ varying from 0.75 to 1.00. Although the stand-alone M5P model performed well for river flow predictions, the M5P ensembles with BA, DA, AR, V, ICO, RS, and ROF resulted in 2.3 %, 2.92 %, 1.99 %, 1.33 %, 1.22 %, 1.43 %, and 2.51 % improved predictive performance for *Q*_*t*_ (based on the *R*^*2*^ indicator), 0.15 %, 22.64 %, 9.43 %, 10 %, 9.43 %, 16 %, and 21.5 % for *Q*_*t+1*_, and 3.2 %, 12 %, 4 %, 2 %, 6 %, 3.6 % and 10 % for *Q*_*t+2*_ forecasting.

Further detailed statistical error indexes were determined for the stand-alone and ensemble M5P models, including RMSE, NSE, PBIAS, and RSR quantitative metrics. Fig. 7 shows that the DA-M5P ensemble algorithm outperforms other models across all three forecasting time steps (i.e., *Q*_*t*_, *Q*_*t+1*_, and *Q*_*t+2*_), followed by ROF-M5P, BA-M5P, AR-M5P, AR-M5P, RS-M5P, V-M5P, ICO-M5P, and stand-alone M5P. All the ensemble models developed in this study have improved the prediction performance of the initial stand-alone M5P model. It was shown that by increasing the forecasting time-step of the river flow (i.e. from *Q*_*t*_ to *Q*_*t+2*_), the model's performance was decreased, and higher uncertainty was found in the predictions. Although stand-alone and ensemble M5P models yield robust predictions, the future forecasting capabilities of these models are still associated with relatively high uncertainty.

**Fig. 8:**
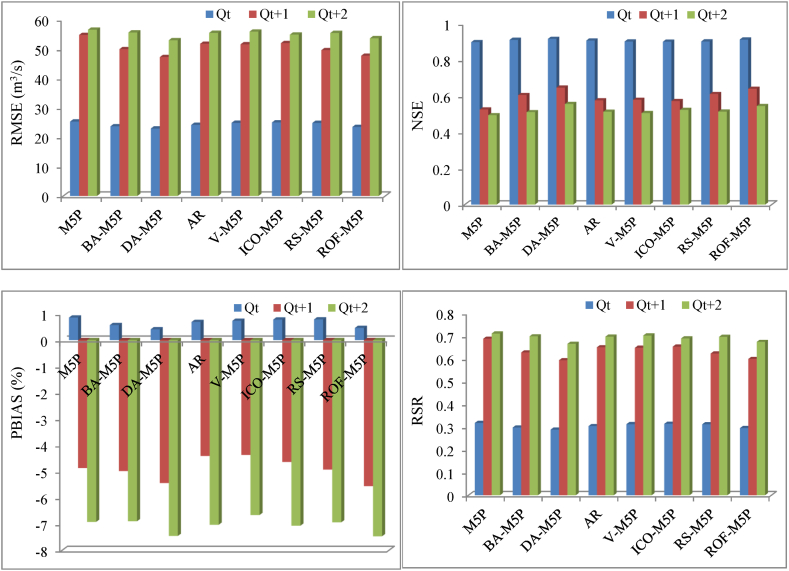
Statistical performance evaluation metrics for the stand-alone and ensemble M5P models.

Overall, the results of the statistical error indexes in Fig. 8 (i.e., NSE, PBIAS, and RSR) confirm the appropriateness and robustness of all developed models for *Q*_*t*_ prediction. For *Q*_*t+1*_ forecasting, all the developed models showed satisfactory performance in terms of NSE metric, whilst good performance for RSR and very good performance for PBIAS were achieved. The performance of the stand-alone M5P model for the PBIAS index was satisfactory. For *Q*_*t+2*_ forecasting, the NSE and RSR criteria described all the developed ensemble models as satisfactory. However, the stand-alone M5P model forecasting for *Q*_*t+2*_ was cconsidered unsatisfactory for the NSE and RSR, while based on the PBIAS metric, all the developed models exhibit very good performance.

The results of *a*_20_ reliability assessment for all developed models at three horizons are presented in Table 8. According to the results, the reliability assessment for each model at the same horizon is very close. However, the reliability sharply decreases for future forecasting.

**Table 8:** Comparison of predictive models’ reliability assessment for river flow forecasting.

**Model**	***Q***_***t***_	***Q***_***t+1***_	***Q***_***t+2***_
M5P	0.526	0.260	0.265
BA-M5P	0.528	0.280	0.291
DA-M5P	0.528	0.277	0.292
AR-M5P	0.528	0.276	0.295
V-M5P	0.527	0.276	0.291
ICO-M5P	0.527	0.280	0.292
RS-M5P	0.525	0.278	0.292
ROF-M5P	0.528	0.281	0.293

**Fig. 9:**
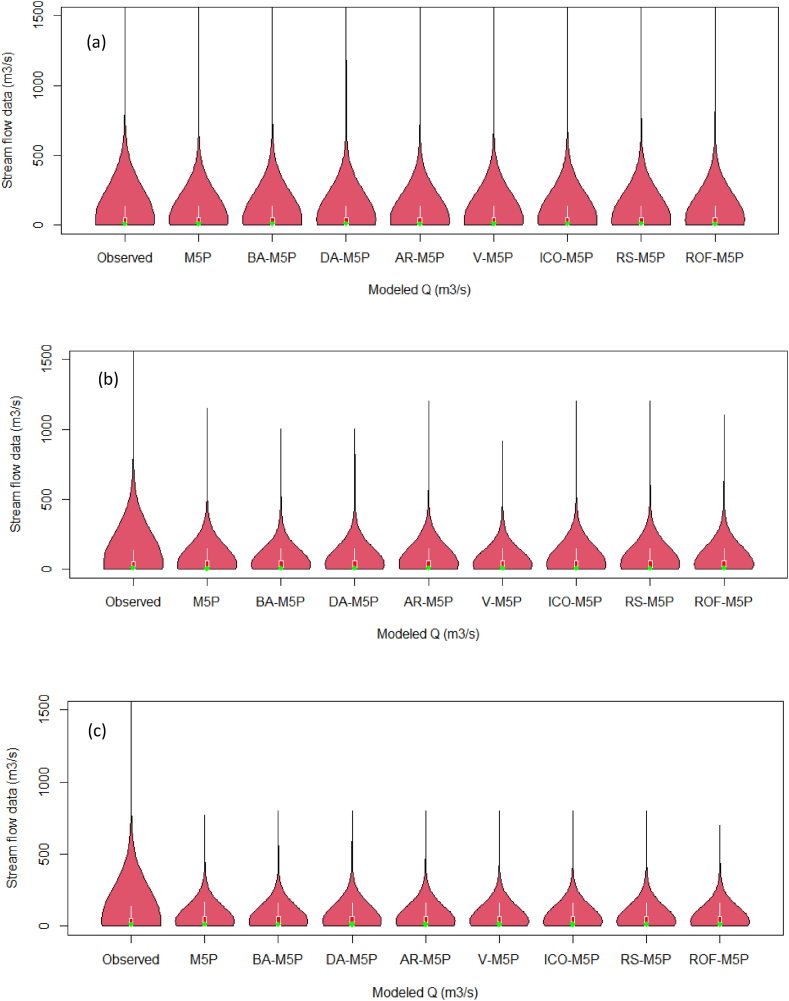
Violin plot of predicted and observed river flow during the testing phase for: (a) *Q*_*t*_, (b) *Q*_*t+1*_*,* and (c) *Q*_*t+2*_.

For *Q*_*t*_ prediction, all developed models exhibit a high predictive power for estimating maximum, quartiles, median, and minimum values, while ROF-M5P close aligns with the observed data (Fig. 9a). However, for *Q*_*(t+1)*_, none of the applied models can accurately forecast the maximum value, with AR-M5P showing greater accuracy compared to other models (Fig. 9b). On the other hand, for *Q*_*(t+2)*_, ROF-M5P exhibits lower accuracy (Fig. 9c).

**Fig. 10:**
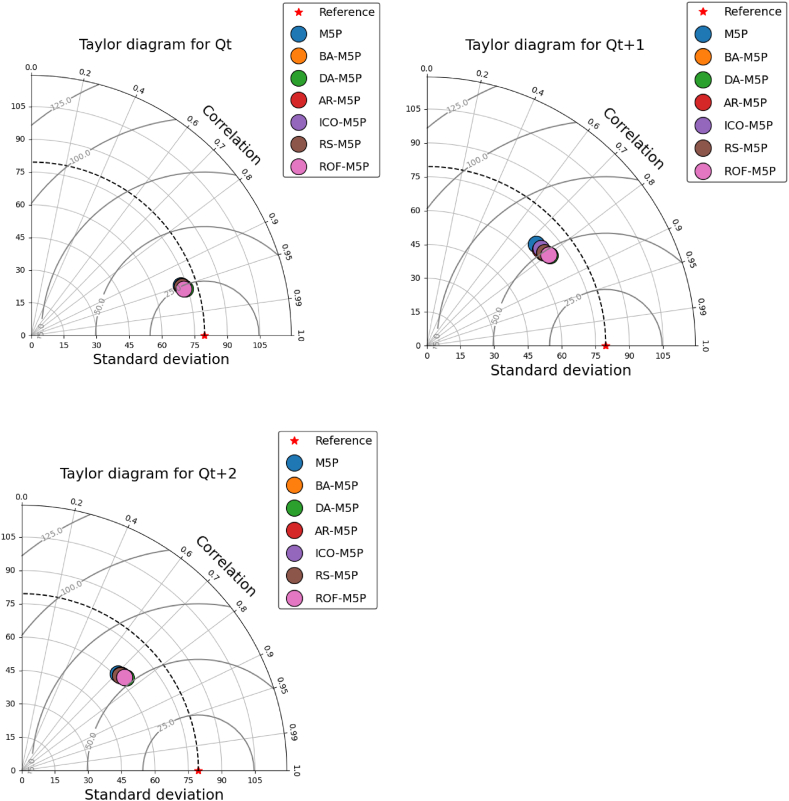
Taylor diagram comparing the performance of stand-alone and ensemble M5P predictive models for forecasting *Q, Qt*_*+1*_*,* and *Q*_*t+2*_.

Fig. 10 presents the Taylor diagram for model evaluation and comparison. The results indicate that most of the applied models exhibit similar predictive power for *Q*_*t*_ predictions. However, the accuracy diverges for future forecasts. Specifically, the ROF-M5P model demonstrates superior accuracy for *Q*_*t+1*_ and *Q*_*t+2*_ forecasting, outperforming the other models. In contrast, the standalone M5P model reveals the lowest performance in these future forecasting scenarios.

**Fig. 11:**
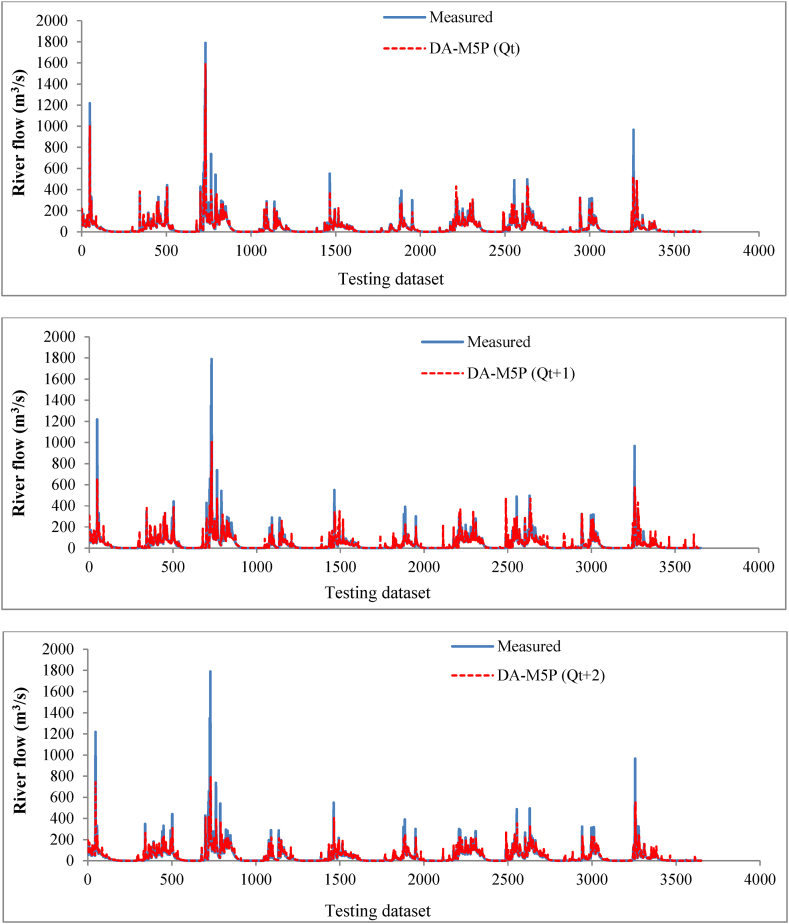
Time series of observed and simulated *Q*_*t*_*, Q*_*t+1*,_ and *Q*_*t+2*_ for testing dataset using DA-M5P ensemble model.

The time-series variation graph for the DA-M5P model, as the optimum algorithm with the highest performance for *Q*_*t*_*, Q*_*t+1*,_ and *Q*_*t+2*_ is presented in Fig. 11. The results demonstrate robust alignment between the DA-M5P predictions and the measured river flow. Although the DA-M5P model underpredicts the maximum *Q*_*t*_ values compared to the same measured values, the model performance in approximating the extreme values is reasonably well. For *Q*_*t+1*_ and *Q*_*t+2*_ forecast, the DA-M5P model has an overall good performance, while for the extreme river flows, the model performance is worsened compared to *Q*_*t*_ prediction results. The results in Fig. 8 demonstrate that increasing the time step of the predictions reduces the forecasting performance.

## 6 Discussion

River flow modeling is a complex hydrological problem influenced by a large range of environmental parameters. An accurate estimate of rive-flow is vital for sustainable water resources management and to mitigate the adverse effects of extreme events and climate stressors in fluvial ecosystems. With the ongoing shifts in climate, the coming decades are anticipated to witness a rise in both the intensity and frequency of extreme weather events, exacerbating the necessity of robust river flow forecasting for surface water, runoff, and flood management.

The choice of parameters and input scenarios for rainfall-runoff (RR) modeling can significantly influence the model's accuracy [23]. The majority of studies in the literature only considered river flow lags, and in some cases, rainfall data together with river flow were incorporated to estimate river flows. The present time river flow is influenced by prior rainfall events, which reach the outlet with lags due to the time of concentration; thus, both rainfall and its lags are critical for accurate modeling. Additionally, river flow is a function of previous discharge, which helps machine learning (ML)-based models recognize trends in the data more accurately.

In recent years, successful implementation of AI techniques for river flow and rainfall-runoff modeling has been reported [81]. The AI techniques can approximate the existing functions between variables involved without prior information about the physics of the problem [82,83]. While AI approaches provide computationally efficient and relatively robust predictions, the uncertainty quantification of the results remains an important problem. Enhancing the performance of AI models for now-casting and near future (*t+1* and *t+2*) forecasting is necessary. A comprehensive sensitivity analysis of all the hydrological, geomorphological, and hydrodynamic parameters influencing the river flows can inform the optimal model structure and input parameters for improved forecasting.

This study examined the performance of stand-alone and ensemble M5P models for predicting river flows for a case study of Tuolumne County, California, USA. The M5P model was hybridized by ROF, BA, DA, AR, RS, V, and ICO techniques. Although the MP5 model performed well in predicting river flows, its hybridized versions showed an enhanced predictive performance. Detailed statistical error indexes were determined to compare the models' performance and determine the most appropriate set of input data parameters for accurate river flow forecasting (i.e., *t*+1 and *t*+2). The results obtained in this study show that river flow with lag time is more correlated with river flow at time *t*. However, for the calculation of river flow at time *t*+1, precipitation at time *t* is shown to be the most influential parameter. This finding aligns with the existing knowledge of underlying hydrological processes, and *Q*_*t*_ is the most effective variable in *Q*_*t+2*_ forecasting. The addition of further hydro-meteorological parameters, such as evaporation, has increased the river flow model accuracy. Scenario 10, which incorporates all the dataset parameters, provides a better estimation for discharge at time *t* compared to other scenarios.

The enhanced model performance using ensemble M5P models is more tangible for river flow forecasting at time *t*+1, where a 22.64 % performance improvement was achieved with DA-M5P compared to the stand-alone M5P. A main advantage of the DA algorithm adopted in this study is that it involves a specific number of disjoint samples alternated with bootstrap samples to obtain the base. The BA and DA techniques are essentially resampling ensemble methods with the ability to generate and combine a variety of classifiers using the same learning algorithm for the base classifier. There are certain advantages to disjoint routes over non-disjoint routes, including a higher fault tolerance. The total resources available in non-disjoint routes are often less than those in disjoint routes, as non-disjoint routes involve shared nodes or links. In contrast, disjoint routes maximize aggregate resources by ensuring that neither nodes nor links are shared between the paths. Non-disjoint routes can be adversely affected by a single link or node failure, while the failure of a link in a disjoint route will only cause a single route to fail [84]. Overall, the ensemble technique benefits from the integration of two models, resulting in higher performance compared to standalone models. Additionally, since the input scenario is the same for all developed models, the differences in their performance can be attributed to variations in the model structure and predictive ability.

All models showed high and accurate predictions for *Q*_*t*_ (NSE>0.9), while for *Q*_*t+1*_ and *Q*_*t+2*_, the performance decreased to around 0.64 and 0.55, respectively. This indicates that forecasting future river flows comes with higher uncertainty, while predicting current river flow (i.e., *Q*_*t*_) is relatively easier, and generally, machine learning models outperform physically-based and conceptual models. Previous studies have also shown the superiority of machine learning models in river flow prediction. Rezaie-Balf et al. [85] used model tree (MT), artificial neural network (ANN), and multivariate adaptive regression splines (MARS) to predict river flow in the Tajan catchment in Iran and found that the MT model (R^2^ = 0.80) performed better than ANN (R^2^ = 0.78) and MARS (R^2^ = 0.79). Khosravi et al. [13] compared various machine learning models, such as BA-M5P, BA-RF, RF, M5R, with SWAT and IHACRES models and reported that all developed machine learning models had higher performance than SWAT and IHACRES. Khosravi et al. [36] applied BA-REPT, RC-REPT, RS-REPT, and AR-REPT for river flow prediction in the Kurkursar watershed in northern Iran and reported that the NSE of the developed models varied between 0.65 and 0.84. Ha et al. [11] compared the prediction accuracy of river flow based on deep learning neural networks with El Niño-Southern Oscillation, and in all cases, the R^2^ was reported lower than 0.85. The results indicate that using only precipitation as input does not robustly simulate river flows, potentially leading to lower performance of empirical models in predicting river flows. In this study, scenarios were developed by considering 1- to 3-day lags of river flow, and precipitation, parameters. For floodplain management, longer lead periods, such as weekly and monthly, should be considered. Incorporating detailed hydro-meteorological parameters in the model structure could further enhance predictions.

### 6.1 Limitations and directions for future research

The analysis of case study data demonstrates the appropriateness and robustness of the proposed M5P-based predictive modeling framework developed in this study, as a reliable and cost-effective tool for river flow forecasting. Future research can extend the proposed methodological framework to various hydro-climatic regions by incorporating a greater number of hydro-meteorological stations. Additionally, it is recommended to investigate the impact of pre-processing and data decomposition techniques, such as Wavelet Transform (WT) and Gaussian Filter (GF), on prediction accuracy. A comparative analysis of prediction performance using reanalysis data versus satellite and remote sensing-based data also holds significant potential and warrants further exploration.

One limitation of the current study is the reliance on offline data. To enhance model prediction accuracy and applications for environmental management, future studies should utilize online and more extensive datasets. Moreover, it is crucial to consider the profound effects of changes in data characteristics induced by climate change and anthropogenic activities [86,87], on river flow forecasting. Incorporating these factors will help refine predictions and improve the adaptability of the models to evolving environmental conditions.

## 7 Conclusions

This study developed a robust ML-based predictive modeling framework for accurate river flow estimation (*Q*_*t*_) and short-term forecasting (*Q*_*t+1*_ and *Q*_*t+2*_). We investigated the efficacy of the stand-alone M5 Prime (M5P) algorithm and its ensemble variants, including Bootstrap Aggregation (BA), Disjoint Aggregating (DA), Additive Regression (AR), Vote (V), Iterative classifier optimizer (ICO), Random Subspace (RS), and Rotation Forest (ROF) for predicting river flow. The proposed modelling framework was successfully applied to the Tuolumne River in California, USA, using readily available environmental sensing and hydrometry data.

Models were constructed using precipitation (*P*_*t*_), evaporation (*E*_*t*_), and river flow (*Q*_*t*_) variables, incorporating both current and lagged values. Ten different combinations of these variables were analyzed using autocorrelation function (ACF), partial autocorrelation function (PACF), and Spearman Correlation Coefficient (SCC) to optimize input scenarios. Results indicate that *Q*_*t-1*_ is the most influential predictor for both *Q*_*t*_ and *Q*_*t+2*_, followed by *Q*_*t-2*_, *P*_*t*_, *P*_*t-1*_, *Q*_*t-3*_, *P*_*t-2*_, *P*_*t-3*_, *E*_*t*_, *E*_*t-1*_, *E*_*t-2*_, and *E*_*t-3*_, respectively. It was shown that for *Q*_*t+1*_ forecasting, *P*_*t*_ emerged as the most significant variable. Overall, river flow with lag time proved to be more impactful on predictions than precipitation, regardless of lag time. The evaporation variable, on the other hand, had the least effect on the model performance.

Interestingly, relying solely on the variable with the highest SCC (i.e. *Q*_*t-1*_) did not achieve satisfactory predictive accuracy, suggesting that SCC alone is insufficient for optimizing model performance. Moreover, increasing the forecast horizon from 1 to 3 days led to a decline in predictive accuracy. The study also found that the most effective input combinations vary across *Q*_*t*_, *Q*_*t+1*,_ and *Q*_*t+2*_ forecasting horizons. While all input variables, including those with lower influence, were necessary for robust *Q*_*t*_ predictions, the inclusion of low-impact variables in *Q*_*t+1*_ and *Q*_*t+2*_ models did not significantly enhance performance. Among the models tested, the DA-M5P ensemble algorithm consistently outperformed others across all forecasting horizons (i.e. *Q*_*t*_, *Q*_*t+1*_, and *Q*_*t+2*_), followed by ROF-M5P, BA-M5P, AR-M5P, AR-M5P, RS-M5P, V-M5P, ICO-M5P, and the stand-alone M5P model. Ensemble methods enhanced the predictive performance of the M5P model by 1.2 %–22.6 %, highlighting their efficacy and potential for improving hydrological forecasting. Future research should investigate the impact of advanced pre-processing and data decomposition techniques on model accuracy. Additionally, a comparative analysis of prediction performance using reanalysis data versus satellite and remote sensing-based data are recommended to further validate and enhance the reliability of the proposed predictive modeling framework.

## Funding

This work was supported by Korea Environment Industry & Technology Institute (KEITI) through R&D Program for Innovative Flood Protection Technologies against Climate Crisis Program (or Project), funded by Korea Ministry of Environment (MOE)(RS-2023-00218873). SA acknowledges support form Natural and Environmental Research Council (NE/S007350/1) and the Scientific Computing Research Technology Platform (SCRTP) at the University of Warwick.

## Data availability

Data will be made available on request.

## Ethics approval

Not applicable.

## Consent to participate

Not applicable.

## Consent for publication

Not applicable.

## CRediT authorship contribution statement

**Khabat Khosravi:** Writing – review & editing, Writing – original draft, Software, Methodology, Formal analysis, Data curation, Conceptualization. **Nasrin Attar:** Writing – original draft, Conceptualization. **Sayed M. Bateni:** Writing – review & editing, Conceptualization. **Changhyun Jun:** Writing – review & editing, Conceptualization. **Dongkyun Kim:** Writing – review & editing, Conceptualization. **Mir Jafar Sadegh Safari:** Writing – original draft. **Salim Heddam:** Writing – original draft. **Aitazaz Farooque:** Writing – review & editing. **Soroush Abolfathi:** Writing – review & editing.

## Declaration of competing interest

The authors declare that they have no known competing financial interests or personal relationships that could have appeared to influence the work reported in this paper.
